# Polymorphism in the gene encoding toll-like receptor 10 may be associated with asthma after bronchiolitis

**DOI:** 10.1038/s41598-017-03429-x

**Published:** 2017-06-07

**Authors:** Sari Törmänen, Matti Korppi, Johanna Teräsjärvi, Juho Vuononvirta, Petri Koponen, Merja Helminen, Qiushui He, Kirsi Nuolivirta

**Affiliations:** 1 Tampere University Hospital, Tampere, Finland; 2 Centre for Child Health Research, Tampere University and University Hospital, Tampere, Finland; 3 Department of Medical Microbiology and Immunology, Turku University, Turku, Finland; 4 Department of Medical Microbiology, Capital Medical University, Beijing, China; 5 Department of Paediatrics, Seinäjoki Central Hospital, Seinäjoki, Finland

## Abstract

Toll-like receptors (TLRs) recognise microbes that contribute to the severity of bronchiolitis and the subsequent risk of asthma. We evaluated whether post-bronchiolitis asthma was associated with polymorphisms in the *TLR3* rs3775291, *TLR4* rs4986790, *TLR7* rs179008, *TLR8* rs2407992, *TLR9* rs187084, and *TLR10* rs4129009 genes. The gene polymorphisms were studied at the age of 6.4 years (mean) in 135 children hospitalised for bronchiolitis in infancy. The outcome measure was current or previous asthma. Current asthma was more common (30%) in children with the variant AG or GG genotype in the *TLR10* rs4129009 gene versus those who were homozygous for the major allele A (11%) (p = 0.03). The adjusted odds ratio (aOR) was 4.30 (95% CI 1.30–14.29). Asthma ever was more common (34.6%) in girls with the *TLR7* variant AT or TT genotype versus those who were homozygous for the major allele A (12.5%) (p = 0.03). The adjusted OR was 3.93 (95% CI 1.06–14.58). Corresponding associations were not seen in boys. There were no significant associations between *TLR3*, *TLR4*, *TLR8*, or *TLR9* polymorphisms and post-bronchiolitis asthma. Polymorphism in the *TLR10* gene increases and in the *TLR7* gene may increase the risk of asthma in preschool-aged children after infant bronchiolitis.

## Introduction

Bronchiolitis in infancy increases the risk of subsequent wheezing and childhood asthma^1^. Although many asthma risk factors, such as asthma in parents, atopy or eosinophilia in children, and rhinovirus aetiology of bronchiolitis^2^, are well documented, predicting the outcome of an individual patient is not possible. Innate immunity, which is highly regulated by genes, plays a crucial role in both infection and inflammation^3^. The development of asthma is a complicated and multifactorial process in which genes interact with the environment^4^. In early life, the Th2-dominated immune responses shift towards Th1-dominated responses^5^, but among genetically susceptible individuals, environmental factors like viruses may lead to the persistence of Th2-dominated immunity and to subsequent atopy and asthma^6^.

Toll-like receptors (TLRs) are pattern-recognising proteins that, after recognising foreign material like microbes, are able to trigger the production of mediators of innate immunity and, subsequently, after complex signalling processes, the development of adaptive immune responses^7, 8^. TLRs 1, 2, 4, 5, 6, and 10 are located on the cell surface, whereas TLRs 3, 7, 8, and 9 are located inside the cells^9^, recognising microbial components after endocytosis. TLR1, TLR2, TLR6, and TLR10 comprise the TLR2 subfamily, and *TLR1*, *TLR2*, *TLR6*, and *TLR10* gene polymorphisms seem to play a role in susceptibility to asthma, atopic eczema, and allergic rhinitis^10–12^. TLR3 recognises double-stranded viral ribonucleic acid (RNA), and, in mice, TLR3 activation by viruses combined with allergen inhalation resulted in allergic airway disease^13^. TLR4 recognises bacterial lipopolysaccharides and the F glycoprotein of the respiratory syncytial virus (RSV)^14^. TLR7 and TLR8, which are regulated by genes located in the X chromosome, recognise single-stranded viral RNA^15^. An American study found that TLR7 contributed to human airway relaxation via the production of nitric oxide^16^. There is evidence that polymorphisms in the *TLR7* and *TLR8* genes are associated with susceptibility to asthma and related atopic disorders^15^ and to susceptibility to respiratory viral infections^17^. Signalling via TLR7 and TLR9 affects the function of eosinophils, engendering a link between viral infection and allergic exacerbations^18^. Although TLR10 is a pattern-recognition receptor without known ligand specificity, it has shown to be a modulatory receptor with mainly inhibitory properties^19^.

We have prospectively followed 166 children who were hospitalised for bronchiolitis at less than 6 months of age^2^. At 5 to 7 years of age, 127 of the children attended a clinical control visit, and questionnaire data were available for another 39 children^2^. We have previously studied the *TLR1* rs5743618, *TLR2* rs5743708, and *TLR6* rs5743810 polymorphisms and reported their associations with post-bronchiolitis asthma at preschool age^10^. The present study was carried out to complement this exploratory study series by evaluating whether the *TLR3* rs3775291, *TLR4* rs4986790, *TLR7* rs179008, *TLR8* rs2407992, *TLR9* rs187084, and *TLR10* rs4129009 polymorphisms are associated with post-bronchiolitis asthma. The aim of this study was to compare these polymorphisms between children with and without current asthma, current atopic dermatitis, or current allergic rhinitis at preschool age, or with and without asthma ever combining current and previous asthma in children hospitalised for bronchiolitis in infancy.

## Results

The mean age of the 135 patients was 6.4 years at the control visit, and 51% were males. Asthma ever was present in 37 patients (27.4%), current asthma in 18 (13.3%), atopic dermatitis in 46 (34.1%), and allergic rhinitis in 39 (28.9%). The genotypes and minor allele frequencies (MAF) and population data on the MAFs are listed in Table 1. The MAFs of the cases and the Finnish population MAF data^20^ did not differ substantially in terms of *TLR3* rs3775291, *TLR4* rs4986790, *TLR7* rs179008, *TLR8* rs2407992, *TLR9* rs187084, or *TLR10* rs4219009 genes.

**Table 1:** Genotypes and minor allele frequencies of genes encoding toll-like receptors 3, 4, 7, 8, 9, and 10 in 135 children hospitalised for bronchiolitis and in the Finnish population.

SNP (Major > Minor)	Major/Major	Major/Minor	Minor/Minor	MAF	FIN
*TLR3* rs3775291 (C > T)	0.46	0.41	0.13	0.33	0.33
*TLR4* rs4986790 (A > G)	0.84	0.16	0	0.08	0.12
*TLR7* rs179008 (A > T)	0.61 (girls)	0.35 (girls)	0.04 (girls)	0.22 (girls and boys)	0.31 (girls and boys)
	0.79 (boys)	0	0.21 (boys)		
*TLR8* rs2407992 (G > C)	0.35 (girls)	0.48 (girls)	0.17 (girls)	0.45 (girls and boys)	0.36 (girls and boys)
	0.79 (boys)	0	0.49 (boys)		
*TLR9* rs187084 (T > C)	0.32	0.43	0.25	0.46	0.45
*TLR10* rs4129009 (A > G)	0.84	0.15	0.01	0.08	0.08

MAF = minor allele frequency, FIN = Finnish MAFs as in ref. 20. N = 135 for *TLR3*, *TLR4*, *TLR7*, and *TLR8*; N = 134 for *TLR9* and *TLR10*.

The *TLR3* genotype was wild (CC) in 45.9% and variant (TC or TT) in 54.1% of the cases. The *TLR4* genotype was wild (AA) in 83.7% and variant (AG) in 16.3% of the cases. The *TLR9* genotype was wild (TT) in 32.1% and variant (TC or CC) in 67.9% of the cases. There were no significant associations between the *TLR3*, *TLR4*, or *TLR9* genotypes and asthma ever, current asthma, current atopic dermatitis, or current allergic rhinitis (Table 2).

**Table 2:** Genotypes of *TLR3* rs3775291, *TLR4* rs4986790, *TLR9* rs187084, and *TLR10* rs4129009 encoding genes in relation to asthma and allergy at preschool age in 135 former bronchiolitis patients.

	Asthma ever	Current asthma	Current atopic dermatitis	Current allergic rhinitis
***TLR3*** **rs3775291** N = 135	N = 37	N = 18	N = 46	N = 39
Wild CC N = 62 (%)	19 (30.6)	8 (12.9)	20 (32.3)	16 (25.8)
Variant CT, TT N = 73 (%)	18 (24.7) p = 0.45	10 (13.7) p = 0.55	26 (35.6) p = 0.72	23 (31.5) p = 0.57
***TLR4*** **rs4986790** N = 135	N = 37	N = 18	N = 46	N = 39
Wild AA N = 113 (%)	31 (27.4)	16 (14.2)	39 (34.5)	34 (30.1)
Variant AG, GG N = 22 (%)	6 (27.3) p = 1.00	2 (9.1) p = 0.74	7 (31.8) p = 1.00	5 (22.7) p = 0.61
***TLR9*** **rs187084** N = 134	N = 37	N = 18	N = 46	N = 39
Wild TT N = 43 (%)	11 (25.6)	4 (9.3)	10 (23.3)	13 (30.2)
Variant TC, CC N = 91	26 (28.0)p = 0.44	14 (15.4) p = 0.25	36 (40.0) p = 0.06	26 (28.6) p = 0.50
***TLR10*** **rs4129009** N = 134	N = 37	N = 18	N = 46	N = 39
Wild AA N = 113 (%)	28 (24.8)	12 (10.6)	39 (34.5)	31 (27.4)
Variant AG, GG N = 21 (%)	9 (42.9) p = 0.08	6 (28.6) p = 0.03	7 (33.3) p = 0.57	8 (38.1) p = 0.23

In females, the *TLR7* genotype was wild (AA) in 60.6% and variant (AT or TT) in 39.4% of the cases. In males, allele A was present in 79.4% and allele T in 20.6%. Asthma ever was present in 34.6% of the girls who had the variant AT or TT genotype compared to 12.5% of those who were homozygous for the major allele A (p = 0.03) (Table 2). The odds ratio (OR) adjusted for age was 3.71 (95% confidence intervals [CI] 1.08–12.77). This association was significant in logistic regression adjusted first for early-life risk factors, and then separately for current confounders (data not shown). The association remained significant in logistic regression adjusted for age, early-life risk factors, and current confounders in the same model (OR 3.93, 95% CI 1.06–14.58). The corresponding figures in boys were 33.3% (allele A present) and 37.5% (allele T present) (p = 1.00). There were no significant associations between the *TLR7* genotypes and current asthma, current atopic dermatitis, or current allergic rhinitis in either girls or boys (Table 3).

**Table 3:** Genotypes of *TLR7* rs179008 and *TLR8* rs2407992 encoding genes in relation to asthma and allergy at preschool age in 135 former bronchiolitis patients.

	Asthma ever	Current asthma	Current atopic dermatitis	Current allergic rhinitis
***TLR7*** **rs179008 Females** N = 66	N = 14	N = 8	N = 23	N = 17
Wild AA N = 40 (%)	5 (12.5)	3 (7.5)	15 (37.5)	10 (25.0)
Variant AT, TT N = 26 (%)	9 (34.6) p = 0.03	5 (19.2) p = 0.25	8 (30.8) p = 0.61	7 (27.0) p = 1.00
***TLR8*** **rs2407992 Females** N = 66	N = 14	N = 8	N = 23	N = 17
Wild GG N = 23 (%)	5 (21.7)	2 (8.7)	8 (34.8)	5 (21.7)
Variant GC, CC N = 43 (%)	9 (20.9) p = 1.00	6 (14.0) p = 0.70	15 (32.6) p = 1.00	12 (26.1) p = 0.77
***TLR7*** **rs179008 Males** N = 68	N = 23	N = 10	N = 23	N = 22
Wild, allele A present N = 54 (%)	18 (33.3)	7 (13.0)	19 (35.2)	19 (35.2)
Variant, allele T present N = 14 (%)	5 (37.5) p = 1.00	3 (21.4) p = 0.68	3(21.4) p = 0.76	3 (21.4) p = 0.36
***TLR8*** **rs2407992 Males** N = 69	N = 23	N = 10	N = 23	N = 22
Wild, allele G present N = 35 (%)	11 (31.4)	6 (17.1)	12 (34.3)	13 (37.1)
Variant, allele C present N = 34 (%)	12 (35.3) p = 0.80	4 (11.8) p = 0.73	11 (32.4) p = 1.00	9 (26.5) p = 0.44

*TLR7* and *TLR8* are presented separately for females and males. One test result of a *TLR7* male presented AT heterozygote and was deleted from the analyses.

In females, the *TLR8* genotype was wild (GG) in 34.8% and variant (GC or CC) in 65.2% of the cases. In males, allele G was present in 50.7% and allele C in 49.3%. There were no significant associations between the *TLR8* genotypes and asthma ever, current asthma, current atopic dermatitis, or current allergic rhinitis in either girls or boys (Table 3).

The *TLR10* genotype was wild (AA) in 84.3% and variant (AG or GG) in 16.7% of the cases. Current asthma was present in 30.0% of the children who had the variant AG or GG genotype compared to 10.6% of those who were homozygous for the major allele A (p = 0.03) (Table 2). The OR adjusted for age and gender was 3.74 (95% CI 1.19–11.78). This association was significant in logistic regression adjusted first for early-life risk factors, and then separately for current confounders (data not shown). The association remained significant in logistic regression adjusted for age, gender, early-life risk factors, and current confounders in the same model (OR 4.30, 95% CI 1.30–14.29). There were no statistically significant associations between *TLR10* gene polymorphisms and asthma ever, current atopic dermatitis, or current allergic rhinitis (Table 2).

## Discussion

There were three main results in our study on the association of TLRs with asthma at 5 to 7 years of age after hospitalisation for bronchiolitis at less than 6 months of age. Firstly, current asthma was more common in children who had the variant *TLR10* rs4129009 genotype. Secondly, asthma ever was more common in girls who had the variant *TLR7* rs179008 genotype. Both findings were robust to adjustments with known early-life risk factors for asthma as well as with current confounders at the age of 5 to 7 years. However, *TLR10* and *TLR7* gene polymorphisms had no significant associations with current allergy. And thirdly, there were no significant associations between *TLR3* rs3775291, *TLR4* rs4986790, *TLR8* rs2407992, or *TLR9* rs187084 polymorphisms and earlier or current asthma or allergy.

TLRs play a pivotal role in promoting and controlling innate immune responses. Functional gene polymorphism alters the amino acid structure of the receptor, as has been shown in the cases of *TLR3* rs3775291 (Leu > Phe), *TLR4* rs4986790 (Asp > Gly), *TLR7* rs179008 (Glu > Leu), *TLR9* rs187084 (Arg > Trp), and *TLR10* rs4219009 (Ile > Leu) gene polymorphisms^21, 22^. The consequence of the mutation is dependent on its location. Mutation in the extracellular domain of the receptor may further lead to an altered binding affinity and subsequent immune response^23^, whereas mutation in the cytoplasmic TIR (toll/interleukine-1 receptor) domain, as in the case of *TLR10* rs4219009, may result in an altered downstream signalling, despite normal binding^12, 24^. Although polymorphism in the *TLR8* rs2407992 (2040 C/G) does not change the amino acid (651Leu > Leu), it can potentially affect TLR8 splicing^15^.

TLR10 is a modulatory pattern-recognition receptor with mainly inhibitory properties, and it is able to reduce TLR2 responses by increasing the production of anti-inflammatory IL-1Ra^19^. Further, a recent meta-analysis revealed that polymorphisms of the IL-1Ra encoding genes were associated with asthma, especially in Caucasian populations^25^. Our finding that the *TLR10* gene polymorphism was associated with current asthma is in accordance with these observations. The genetic variation in *TLR10* rs4129009, which was also determined in the present study, was associated with asthma risk in two independent samples from the USA^26^. In addition, in a Canadian–Australian study, a weak association was observed between another *TLR10* polymorphism (rs11096957) and atopic asthma^27^.

In a large German study, a protective effect of genetic variants on atopic asthma was identified in the TLR2-associated heterodimer network consisting of TLR1, TLR6, and TLR10^12^. Corresponding findings in the genes encoding TLR1, TLR2, and TLR6 were also seen in the present post-bronchiolitis cohort, but the direction of the effect was opposite^10^. The variant genotype in the *TLR1* gene was associated with asthma during the first 6 years of life, and asthma was present in only two children with the wild genotype in all three polymorphisms^10^. In the most recent study from this cohort^28^, polymorphism of *TLR6* was associated with bronchial hyper-reactivity, and if all of the four genes including *TLR10* presented with the wild genotype, exercise-induced responses in resistance at 5HZ by impulse oscillometry were significantly smaller than in those with one or more variant genotypes. These findings are in accordance with our current observations stressing the role of the variant *TLR10* genotypes in the emergence of post-bronchiolitis asthma. The differences between the German^12^ and Finnish cohorts may be due to different asthma phenotypes, allergic asthma in the German study, and post-bronchiolitis asthma in the current study.

The German study reported that primary cells derived from carriers of protective *TLR1*, *TLR6*, and *TLR10* variants showed augmented inflammatory responses, increased Th1 cytokine expression, and reduced Th2-associated IL-4 production after specific stimulation^12^. The suppressed secretion of allergy-related cytokines, like IL-4, IL-15, and IL-13, seems to be associated with asthma phenotypes not related to allergy^29^.

We found preliminary evidence that the role of the *TLR7* gene in asthma may differ between girls and boys, which may be explained by the situation of the *TLR7* rs179008 in the X chromosome^30^. A recent experimental study reported that a *TLR7* gene defect and early pneumovirus infection in mice interacted with each other, first leading to a severe bronchiolitis-like disease, then to Th2-dominated immunity, and finally to an asthma-like pathology^31^. A Danish study revealed that *TLR7* rs179008 polymorphism was associated with asthma^15^. An American study found that TLR7 was expressed in human airway nerves and contributed to relaxation of the airways via the production of nitric oxide^16^. Therefore, normal TLR7 function may be protective against airway hyper-reactivity and asthma, whereas *TLR7* polymorphism may predispose to asthma, and our observations suggest that the influence is greater in girls than in boys.

Polymorphism in the *TLR8* rs2407992 gene had no association with current or earlier asthma, atopic dermatitis, or allergic rhinitis. This finding contradicts the results of the Danish study^15^, in which the same *TLR8* polymorphism was associated with asthma, atopic dermatitis, allergic rhinitis, and elevated allergen-specific immunoglobulin E. A Swedish case-control study found an association between *TLR8* gene variation and allergic rhinitis in adults^9^.

Polymorphisms in the *TLR3* rs3775291, *TLR4* rs4986790, or *TLR9* rs187084 genes were not associated with current or earlier asthma or allergies. A previous study from the present cohort offered preliminary evidence that the wild *TLR3* rs3775291 genotype increased the risk for repeated post-bronchiolitis wheezing^32^. The negative result of the present study at the preschool age was due, at least partly, to the more beneficial outcome of subjects hospitalised for RSV bronchiolitis compared to subjects hospitalised for rhinovirus bronchiolitis. Herein, subsequent asthma was more common after rhinovirus bronchiolitis than after RSV bronchiolitis^2, 33^. Recent meta-analyses have failed to find any associations between *TLR4* rs4986790 polymorphism and asthma^34^, and between *TLR9* rs187084 polymorphisms and asthma^35^.

There were certain limitations in the present study. The number of patients was relatively small for genetic studies. In addition, blood samples for genetic studies were not available from all cases with sufficient follow-up data available, although we consider the 81% proportion as acceptable. The small number of patients means a risk of type-2 statistical error. On the other hand, we carried out multiple analyses for polymorphisms of six different TLR-encoding genes, which means a risk of type-1 statistical error. We considered our study as an exploratory study aimed at finding preliminary evidence for associations, if present, which needs to be confirmed or rejected in future confirmatory studies. Therefore, and because only two polymorphisms were associated with asthma risk, we did not regard any multiplicity adjustments as necessary^36, 37^. Multivariate logistic regression was used, allowing adjustments with early-life risk factors and current confounding factors, but the power of the study was not sufficient for incorporation of all six polymorphisms in the same model.

The strengths of the present study are the prospective design, relatively long follow-up period of six years, extensive virological test panel available during hospitalisation for bronchiolitis, and careful data collection during bronchiolitis and subsequent control visits. The homogeneity of study populations, as in the present study, is a clear benefit for genetic studies. We consider the revealed significant association between *TLR10* polymorphism and current asthma at preschool age a reliable finding, since the MAF figures were the same, 0.08, in both cases and population-based controls from the FIN data^20^. Since *TLR7* and *TLR8* genes are located in the X chromosome, the analyses were carried out separately for both sexes, and indeed, the findings seemed to be different in girls and boys. RSV caused over 70% of the bronchiolitis cases, but subsequent asthma was more common after rhinovirus bronchiolitis than after RSV bronchiolitis^2^. Therefore, the final analyses on the role of *TLR10* rs4129009 were performed with multivariate logistic regression adjusted for age, sex, early-life risk factors, and current risk factors for asthma, including the RSV aetiology of early-life bronchiolitis, and the conclusions did not change.

In conclusion, polymorphism in the *TLR10* gene seems to increase the risk of post-bronchiolitis asthma in preschool-aged children, and polymorphism in the *TLR7* gene seems to increase the risk of post-bronchiolitis asthma in preschool-aged girls. This result was rather strong, since the associations were robust to adjustments for early-life and current risk factors for asthma.

## Methods

The study was conducted at the Department of Paediatrics, Tampere University Hospital, Finland, and the design was previously described^2^. In brief, 166 previously healthy, full-term infants hospitalised for bronchiolitis at less than 6 months of age in 2002–2004 attended a follow-up study in 2008–2010, when the children were 5 to 7 years of age. In infancy, bronchiolitis was defined as an acute lower respiratory illness characterised by rhinorrhea, cough, and diffuse wheezes or crackles^38^. Early-life data were collected by interviewing the parents during hospitalisation using structured questionnaires^2^. This showed that 14.5% of the mothers and 6% of the fathers had asthma, and 29.5% of the children had atopic dermatitis or food allergy. Data on the viral aetiology of bronchiolitis were studied on admission by antigen detection and polymerase chain reaction (PCR), and a viral infection was identified in most cases: RSV in 70.5% and rhinovirus in 12.7%^2^. During the follow-up study, 127 children attended the clinical control visit, and an additional 39 children returned the study questionnaire. The structured questionnaire completed by the parents of both groups consisted of separate questions on wheezing episodes, asthma diagnoses, and use of bronchodilators and inhaled corticosteroids (ICS) for the preceding 12 months. The presence of atopic dermatitis and allergic rhinitis, night cough in the absence of infections, and prolonged cough for more than four weeks were also charted for the preceding 12 months. The subjects of the present study consisted of those 135 children for whom samples for genetic studies are available.

### Definition of asthma

Current asthma was defined as the current use of continuous maintenance medication with ICS for asthma, or suffering from doctor-diagnosed episodes of wheezing, with a prolonged or night cough during the preceding 12 months and with a diagnostic finding in the exercise challenge test (ECT)^2^. The ECT consisted of free running outdoors for 8 minutes and measurements of pre- and post-exercise airway resistance by impulse oscillometry (Jaeger, Master Screen IOS, Höchberg, Germany). An increase in resistance of 35% or more at 5 Hz was considered to be pathological^2, 39^. Previous asthma before the control visit was defined as the previous use of inhaled ICS as continuous or intermittent maintenance medication for asthma^2^. If the child had either previous or current asthma, the term *asthma ever* was used.

Allergic rhinitis was defined as episodes of watery nasal discharge not accompanied by fever or by other symptoms of respiratory tract infection^2^. Atopic dermatitis was defined by doctor-diagnosed eczema and atopy^2^.

### Genotyping

Polymorphisms of *TLR3* rs3775291 (1234 C/T), *TLR4* rs4986790 (1194 A/G), *TLR7* rs179008 **(**171 A/T), *TLR8* rs2407992 (2040 C/G), *TLR9* rs187084 (1486 T/C), and *TLR10* rs4129009 (2322 A/G) were selected due to their evident functional properties. The genotyping of *TLR3* rs3775291 (1234 C/T) was performed by pyrosequencing (PSQ™96MA Pyrosequencer, Biotage, Uppsala, Sweden) using a PSQ™96 Pyro Gold Q96 reagent kit according to the manufacturer’s protocol^32^. The genotyping of TLR4 rs4986790 (299 A/G) was performed by the ABIPRISM 7000 Sequence Detection System (Applied Biosystems, CA)^40^ supplemented later with pyrosequencing (PSQ™96MA Pyrosequencer, Biotage, Uppsala, Sweden) using a PSQ™96 Pyro Gold Q96 reagent kit^41, 42^. The genotyping of *TLR8* rs2407992 (2040 C/G) was performed in the same manner as described for *TLR3* rs3775291 (1234 C/T). For *TLR7* rs179008 (171 A/T), the PCR products were first purified using the QIA quick PCR Purification Kit (Qiagen, Hilden, Germany) according to the manufacturer’s protocol. PCR products with low deoxyribonucleic acid (DNA) content were eluted to 30 µl of elution buffer. After purification, the PCR products were pipetted to a 96-well plate (5 µl) together with *TLR7* rs179008 **(**171 A/T) forward primer (1.6 µl), and the 96-well plate was sent to the Institute for Molecular Medicine laboratory in Helsinki, Finland, for sequencing, as described recently^28^.

*TLR9* rs187084 (1486 T/C) genotyping was performed using BspTI restriction enzyme (ThermoFhisher Scientific, Waltham, USA) for digestion of the PCR product^28^. High-resolution melting analysis (HMR) (Roche Diagnostics Light Cycler 480, Basel, Switzerland) was used for genotyping of *TLR10* rs4129009 (2322 A/G), as described previously^28^. There were 135 samples available for genotyping of the *TLR3*, *TLR4*, *TLR7*, and *TLR8*. For the genotyping of the *TLR9* and *TLR10*, 134 samples were available.

### Ethics

The study was carried out in accordance with the approved guidelines of the WMA Declaration of Helsinki. The protocol of the study was approved by the Ethics Committee of the Tampere University Hospital District, Tampere, Finland. Before we enrolled the children, we obtained informed parental consent, including the use of samples for genetic studies on bronchiolitis and asthma risk, during both hospitalisation and the control visit. The personal data of the study subjects were not given to the two laboratories that performed the genetic studies, the National Institute of Health and Welfare, Turku, Finland, or the Institute for Molecular Medicine, Helsinki, Finland.

### Statistics

Statistical analyses were carried out with SPSS version 23.0 software (IBM Corp, NY, USA). The chi-square test and Fisher’s exact test were used, when appropriate, to analyse genotype frequencies between those with and without current asthma, asthma ever, current allergic rhinitis, or current atopic dermatitis. Multivariate analyses were conducted if univariate analyses revealed statistically significant (p < 0.05) associations. Logistic regression was first adjusted for gender and age, and then for gender, age, maternal asthma, and RSV aetiology of bronchiolitis (early-life risk factors), and further for gender, age, and current atopic dermatitis (current confounders). Finally, age, gender, early-life risk factors, and current confounders were included in the same model. The results were expressed as OR and 95% CI. The FINETTI programme was used to evaluate the Hardy–Weinberg equilibrium (HWE) of the studied *TLR3*, *TLR4*, *TLR9*, and *TLR10* alleles, and they were in the HWE. Since *TLR7* and *TLR8* genes are located in the X chromosome, the HWE was not studied, and males and females were analysed separately.
